# Primary prophylaxis of bacterial infections and *Pneumocystis jirovecii* pneumonia in patients with hematologic malignancies and solid tumors: 2020 updated guidelines of the Infectious Diseases Working Party of the German Society of Hematology and Medical Oncology (AGIHO/DGHO)

**DOI:** 10.1007/s00277-021-04452-9

**Published:** 2021-04-13

**Authors:** Annika Y. Classen, Larissa Henze, Marie von Lilienfeld-Toal, Georg Maschmeyer, Michael Sandherr, Luisa Durán Graeff, Nael Alakel, Maximilian Christopeit, Stefan W. Krause, Karin Mayer, Silke Neumann, Oliver A. Cornely, Olaf Penack, Florian Weißinger, Hans-Heinrich Wolf, Jörg Janne Vehreschild

**Affiliations:** 1 Faculty of Medicine and University Hospital Cologne, Department I for Internal Medicine, University of Cologne, Herderstr. 52-54, 50931 Cologne, Germany; 2 German Centre for Infection Research (DZIF), partner site Bonn-Cologne, Cologne, Germany; 3 Department of Medicine, Clinic III – Hematology, Oncology, Palliative Medicine, Rostock University Medical Center, Rostock, Germany; 4 Department of Hematology and Oncology, Clinic for Internal Medicine II, University Hospital Jena, Jena, Germany; 5 Hematology, Oncology and Palliative Care, Klinikum Ernst von Bergmann, Potsdam, Germany; 6 Specialist Clinic for Haematology and Oncology, Medical Care Center Penzberg, Penzberg, Germany; 7 Department I of Internal Medicine, Hematology and Oncology, University Hospital Dresden, Dresden, Germany; 8 Department of Internal Medicine II, Hematology, Oncology, Clinical Immunology and Rheumatology, University Hospital Tübingen, Tübingen, Germany; 9 Department of Medicine 5 – Hematology and Oncology, University Hospital Erlangen, Erlangen, Germany; 10 Medical Clinic III for Oncology, Hematology, Immunooncology and Rheumatology, University Hospital Bonn (UKB), Bonn, Germany; 11 Interdisciplinary Center for Oncology, Wolfsburg, Germany; 12 Faculty of Medicine and University Hospital Cologne, Chair Translational Research, Cologne Excellence Cluster on Cellular Stress Responses in Aging-Associated Diseases (CECAD), University of Cologne, Cologne, Germany; 13 University of Cologne, Faculty of Medicine and University Hospital Cologne, Clinical Trials Centre Cologne (ZKS Köln), University of Cologne, Cologne, Germany; 14 Medical Department for Hematology, Oncology and Tumor Immunology, Charité Universitätsmedizin Berlin, Campus Virchow-Klinikum, Berlin, Germany; 15 Department for Internal Medicine, Hematology/Oncology, and Palliative Care, Evangelisches Klinikum Bethel v. Bodelschwinghsche Stiftungen Bethel, Bielefeld, Germany; 16 Department IV of Internal Medicine, University Hospital Halle, Halle, Germany; 17 Department of Internal Medicine, Hematology/Oncology, Goethe University Frankfurt, Frankfurt am Main, Germany

**Keywords:** Neutropenia, Prophylaxis, Bacterial infections, Resistance, Pneumocystis

## Abstract

Hematologic and oncologic patients with chemo- or immunotherapy-related immunosuppression are at substantial risk for bacterial infections and *Pneumocystis jirovecii* pneumonia (PcP). As bacterial resistances are increasing worldwide and new research reshapes our understanding of the interactions between the human host and bacterial commensals, administration of antibacterial prophylaxis has become a matter of discussion. This guideline constitutes an update of the 2013 published guideline of the Infectious Diseases Working Party (AGIHO) of the German Society for Hematology and Medical Oncology (DGHO). It gives an overview about current strategies for antibacterial prophylaxis in cancer patients while taking into account the impact of antibacterial prophylaxis on the human microbiome and resistance development. Current literature published from January 2012 to August 2020 was searched and evidence-based recommendations were developed by an expert panel. All recommendations were discussed and approved in a consensus conference of the AGIHO prior to publication. As a result, we present a comprehensive update and extension of our guideline for antibacterial and PcP prophylaxis in cancer patients.

**Supplementary Information:**

The online version contains supplementary material available at 10.1007/s00277-021-04452-9.

## Introduction

During recent years, the advent of an increasing amount of targeted drugs and other strategies of personalized medicine has resulted in rapid changes in treatment paradigms for hematologic and oncologic patients. Thus, since the previous version of this guideline many new substances have received approval in high-risk hematology treatment indications (e.g., idelalisib in follicular lymphoma/CLL) while immunological effects and possible risks for opportunistic infections remain poorly examined. However, most new treatment strategies only delay disease progression and most patients still receive conventional anti-cancer chemotherapy at some point of their treatment. As a consequence of immunotherapy- and chemotherapy-related immunosuppression and neutropenia, bacterial infections and *Pneumocystis jirovecii* pneumonia (PcP) are significant causes of morbidity and mortality [1–4]. Most authors define neutropenia as a neutrophil count below 500/μl or < 1000/μl with predicted decline to less than 500/μl within the next 2 days [5, 6]. If no neutrophil count is available or possible, neutropenia can be assumed during leukopenia with leukocytes < 1000/μl.

Although no prospective studies have formally compared possible management strategies, fever during an episode of neutropenia (febrile neutropenia, FN) is generally considered a medical emergency and usually entails rapid initiation of empiric treatment with broad-spectrum antibiotics and in most cases hospitalization. Besides the morbidity and mortality related to FN, the need for antibiotic treatment, treatment side effects, inpatient stay or frequent outpatient visits also impairs quality of life, especially for patients in palliative treatment situations and causes considerable costs [1].

The incidence of febrile neutropenia is highly variable depending on patient-related risk factors as well as type of treatment and ranges from close to 0% (low-intensity treatment for solid tumors) [7] to almost 100% (induction treatment for acute leukemia) [8]. Real-life incidence rates may in general be higher outside the controlled conditions of prospective clinical trials [9].

Many oncologists advocate the use of general preventative strategies aiming at reducing transmission of pathogens via infection control, hygiene, and/or behavioral recommendations, although most infections in neutropenic hosts are usually considered of endogenous origin [10]. Other than that, there are two established pharmacological interventions to reduce incidence of FN: (i) granulocyte colony-stimulating factors (G-CSF), which are the topic of another guideline and (ii) prophylactic antibiotic use [11, 12].

The administration of antibacterial prophylaxis and its impact on patient outcome has been extensively studied. It is generally accepted that this strategy reduces the incidence of febrile events and infections, while survival benefits have only been demonstrated in meta-analyses by including studies from different epochs [13]. Even though prophylactic regimens are generally well tolerated during short-term observation, recent breakthroughs in the understanding of resistance development, spread of multidrug-resistant organisms, and the general interactions between the human host and its commensal microbiota have to be considered in a risk–benefit assessment.

We updated our guideline to give an overview about current evidence on antibacterial prophylaxis strategies and the choice of drugs to prevent bacterial infection and PcP in neutropenic patients. In addition, we revisited patient risk stratification and bacterial epidemiology and considered the current discussion on potential adverse effects caused by antibiotic exposure.

## Methods

This guideline is an update of the 2013 published version [12]. As established, an expert group of hematologists, oncologists, and infectious disease specialists, all of them members of the Infectious Disease Working Party of the German Society of Hematology and Medical Oncology (AGIHO), reviewed and discussed current evidence on antibacterial and PcP prophylaxis. Initially, a literature search of publications from January 2012 to March 2017 was performed and evaluated by subcommittees of two to six experts. The literature review was updated at the time of manuscript finalization (August 2020), which revealed no new breakthrough results necessitating further amendments to the recommendations. All statements and recommendations were discussed in meetings, in telephone conferences, and by electronic correspondence. Recommendations have been approved via expert consensus during the AGIHO plenary meeting on 13 March 2017 and the manuscript was reviewed by all co-authors prior to submission. Evaluation of strength of recommendation and quality of evidence was performed in consistence with other recently updated German and European guidelines (Table 1) [4, 14–16]. The detailed methodology is described in the guideline report.

**Table 1:** Grading

Category, grade	Definition
Strength of recommendation	
A	Strongly supports a recommendation for use
B	Moderate evidence to support a recommendation for use
C	Marginally supports a recommendation for use
D	Supports a recommendation against use
Quality of evidence—level	
I	Evidence from at least one properly designed randomized, controlled trial
II	Evidence from at least one well-designed clinical trial, without randomization; from cohort or case-controlled analytic studies (preferably from > 1 center); from multiple time series; or from dramatic results of uncontrolled experiments
III	Evidence from opinions of respected authorities; based on clinical experience; descriptive case studies; or reports of expert committees
Quality of evidence—index (for level II)	
r	Meta-analysis or systematic review of randomized controlled trials
t	Transferred evidence, that is, results from different patient cohorts, or similar immune status situation
h	Comparator group is a historical control
u	Uncontrolled trial
a	Published abstract (presented at an international symposium or meeting)

This guideline gives recommendations on antibacterial and PcP prophylaxis in neutropenic cancer patients. Recommendations on the diagnosis and empirical treatment of fever of unknown origin during neutropenia can be found in a distinct guideline of the AGIHO [4]. Information on antiviral and antifungal prophylaxis as well as information on infection prevention in patients undergoing allogeneic and autologous stem cell transplantation can also be found in separate AGIHO publications [15–18].

## Patients and risk factors

Reliable identification of hematologic and oncologic patients at risk for febrile neutropenia and infection is a prerequisite to evaluate the need for prophylactic treatment. The chance of infection during neutropenia increases over time; therefore, severity and duration of neutropenia are unequivocal risk factors for the development of infection following chemotherapy [5, 6]. In line with other guidelines [4, 19, 20], we recommend to stratify neutropenic patients into two different risk groups according to the anticipated duration of neutropenia. Patients likely to develop prolonged neutropenia (> 7 days) should be considered as high-risk patients (A-I), whereas patients with estimated duration of neutropenia of seven days or less should not be considered at high risk (A-I), unless additional risk factors are present (B-II) [21, 22] (Table 2).

**Table 2:** Risk factors for bacterial infection during neutropenia

Clinical situation	Intention/recommendation	Intervention	SoR	QoE	References
Patients with prolonged neutropenia (> 7 days)^a^	Identify patients at risk for FN	Consider as high-risk patients	A	I	[5, 19, 23]
Patients with neutropenia > 0 and ≤ 7 days^a^ and significant additional risk factors^b^		Consider as high-risk patients	B	II	[5, 21–23]
Patients with neutropenia ≤ 7 days without additional risk factors		Consider as low-risk patients	A	I	[5, 19, 24]

*SoR* strength of recommendation, *QoE* quality of evidence

^a^This estimation should consider potential G-CSF prophylaxis. If sufficient risk reduction can be achieved by G-SCF, that should be the preferred strategy

^b^See Table 3

Prophylaxis with granulocyte colony-stimulating factors (G-CSF) or granulocyte-macrophage colony-stimulating factors (GM-CSF) should be considered for risk stratification as G-CSF prophylaxis reduces the incidence of FN and bacterial infection by shortening the length of neutropenia [11]. Use of G-CSF is recommended if the anticipated risk for FN is above 20% [11, 23]. There is insufficient clinical evidence to compare clinical outcome for prophylaxis with colony-stimulating factors versus use of antibiotics [23]. If sufficient risk reduction can be achieved by G-CSF administration, that should be the preferred strategy, based on superior tolerability and the absence of selective pressure to bacteria by G-CSF in contrast to antibacterials (B-II).

Numerous studies have assessed risk factors for FN that could potentially guide individual decisions towards antibacterial prophylaxis. Major dilemma is caused by the heterogeneous assessment of discriminating subsets of highly interacting risk factors (e.g., age, comorbidity, performance score, and renal function). This limitation necessitates great caution when comparing results from different studies.

Most established guidelines compile lists of risk factors reported in one or more publications [25–28]. However, this strategy will inevitably lead to an overstatement of the actual risk by disregarding the interactions. We therefore extracted risk factors only from those epidemiological analyses which assessed the risk of FN by multivariable analysis.

Our strategy still revealed a concerning heterogeneity of results between studies. No single patient-related risk factor other than duration of neutropenia and intensity of chemotherapy was consistently identified as an effective predictor of FN. For example, age is generally considered a risk factor for FN and listed as such in all major guidelines [20, 25–27, 29], although 15 of 19 identified studies controlling for confounders with 68,007 of 70,466 patients did not identify age as an independent risk factor in multivariable analysis. The administered dose of chemotherapy has been considered a risk factor for infection during neutropenia [22, 30, 31], while the impact of the chemotherapeutic regimen remains unclear. It has been shown that infection rates differ between treatment regimens [32–34] but not all studies showed statistically significant differences. The risk of febrile neutropenia and infection was shown to be higher during the first than during subsequent cycles of chemotherapy [30, 35–37]. The overall burden of comorbidities, but not low performance scores, seems to be independently associated with the risk for FN; however, no single comorbid condition can be identified as the main driver of FN risk.

Some of the more frequently detected independent factors associated with FN are listed in Table 3. Other additional risk factors, e.g., immunoglobulin deficiency, prior infections, and specific rare comorbidities may play a role in selected patient groups and should be part of individual considerations. However, patients with neutropenia duration of 7 days or less should not be considered at high risk because of one potential risk factor, but by clinical judgment based on comprehensive assessment of patient status and intended treatment. The decision should not only account for clinical risk factors but also patient management aspects (e.g., outpatient/inpatient treatment, estimated compliance, distance from hospital, home environment, and social support).

**Table 3:** Patient-related risk factors

Patient-related risk factors^a^	
Prolonged neutropenia (> 7 days)	
Type and stage of underlying malignancy	
Administered type and dosage of chemotherapy	
First chemotherapy cycle	
Cardiac insufficiency	
Low baseline creatinine clearance	
Low baseline leukocyte count	
Elevated baseline levels of alkaline phosphatase and bilirubin	

^a^Factors that were found independently associated with development of fever during neutropenia in multivariable analyses of either the majority of studies or the majority of patients in studies testing that risk factor

## New anti-cancer agents and antibacterial prophylaxis

The use of new anti-cancer agents, e.g., immune-modulating drugs, antibodies, and molecular targeted agents in clinical routine increases constantly. Many of these agents received fast-track approval by regulatory authorities based on successful phase II–III trials while the impact of such drugs on the incidence of infections is not yet fully determined. Infections have been identified as a potential side effect of several anti-cancer drugs, though exact numbers remain unknown. In the absence of further individual risk factors, we recommend to not classify such patients as high-risk patients of FN when considering antibiotic prophylaxis [38].

To date, antibacterial prophylaxis in non-neutropenic patients is only recommended in those treated with the C5-antibodies eculizumab or ravulizumab without previous meningococcal vaccination (or vaccination < 2 weeks prior to drug administration) to prevent infection by *Neisseria* spp. [39, 40]. Prophylaxis should be performed with penicillin V 250 mg every 12 h, or ciprofloxacin 500 mg daily for at least 4 weeks after complete immunization or until protective titers are documented (A-II_u_), comparable to the situation around surgical splenectomy.

## Spectrum of pathogens in infections of neutropenic patients

While bacteremia is the most commonly identified cause of infection during FN, almost half of the febrile events are ultimately classified as fever of unknown origin (FUO), since no infectious focus can be identified [2]. In cases of microbiologically documented gram-positive bacteremia, the dominant species are typically coagulase-negative staphylococci, α-hemolytic streptococci, and *Staphylococcus aureus*. Sepsis due to gram-negative pathogens is associated with high mortality and often caused by enterobacteria such as *Escherichia coli* or *Klebsiella* spp. and by non-fermenters, in particular *Pseudomonas aeruginosa* [41–44].

In past observations, implementation of antibacterial prophylaxis with activity against gram-negative bacteria was associated with a decrease of gram-negative but an increase of gram-positive bacteria isolated from patients with FN [42, 45]. In the last decade, a new shift towards higher rates of gram-negative pathogens in bacteremia has been described in hematologic patients [43, 44, 46–48]. These observations have recently been confirmed by large multicenter surveillance studies [49–51]. Of note, no significant trend for blood stream infection due to resistant bacteria (methicillin-resistant *Staphylococcus aureus* (MRSA), vancomycin-resistant enterococci (VRE), extended-spectrum β-lactamase (ESBL)-producing *Enterobacteriaceae*) was described. However, the surveillance was performed in patients undergoing hematopoietic stem cell transplantation (HSCT) and the situation for patients with other malignant diseases and other treatment regimens may differ. To better understand the impact of fluoroquinolone (FQ) prophylaxis on local epidemiology, continuous microbiological surveillance is highly recommended. This should not only focus on FQ-resistant pathogens but also on rates of *Clostridioides difficile* infection (CDI) [52–54].

## Resistance development

Bacterial resistance increases globally, which bears two potential implications for prophylactic use of antibiotics: (i) the effectiveness of prophylactic antibiotics may decrease and (ii) the use of broad-spectrum antibiotics in large patient populations without infection undermines current demands for rational anti-infective prescription. The prevalence of colonization due to ESBL *Enterobacteriaceae* increases worldwide, while prior colonization with ESBL-producing pathogens in hematologic/oncologic patients constitutes an important risk factor for subsequent development of ESBL-associated blood stream infections [50, 51, 55]. Concerns about resistance development therefore led some experts to vote against antibacterial prophylaxis in neutropenic patients [19, 24].

The prevalence of resistant pathogens, especially *Escherichia coli* and *Enterococcus faecium*, is higher and increases more rapidly in the hematologic/oncologic compared to other patient populations [47, 48, 56, 57]. Recent studies demonstrated that FQ use may expedite the emergence and colonization with bacterial strains resistant to the administered drug as well as other drugs with genetically linked resistance genes [58, 59]. Studies on discontinuation of antibacterial prophylaxis detected reappearance of gram-negative bacteria in blood culture isolates after FQ restriction, while a decrease in the proportion of FQ-resistant organisms was observed [43, 55, 60]. Yet, an increase of resistance was not uniformly observed across studies during antibiotic prophylaxis [61]. Gudiol et al. described a decrease in FQ resistance (71% versus 37%; *p* < 0.001); an observed concurrent increase in multidrug-resistant gram-negative blood stream infections (BSI) (1% versus 6%, *p* < 0.001) was probably attributable to global changes in the epidemiology of resistant bacteria [43]. Of note, a recently published study demonstrated that the patients’ 30-day mortality was higher when developing bacteremia from FQ-resistant than from FQ-sensitive gram-negatives [62].

We do not generally recommend to use a threshold of FQ resistance among gram-negative bacteria in a given population for instituting FQ prophylaxis (D-III). However, to avoid amplification of resistant clones, we do not recommend to initiate prophylaxis with FQ if colonization of a patient with FQ-resistant gram-negative bacteria is known prior to the onset of prophylaxis (D-II_t,u_). Surveillance of resistance development (A-III) and antibiotic prophylactic efficacy (B-III) should be performed. Considering the local epidemiology, prospective studies are needed for evaluation of FQ prophylaxis in areas with high local rates of FQ resistance among gram-negative bacteria.

## Novel considerations regarding microbiome and resistome

New sequencing techniques have led to an abundance of new insights on host–microbiota interactions which may ultimately in part redefine our understanding of the pathogenesis of many infections and other diseases [63]. Two recent studies by Chong et al. analyzed the impact of prior FQ prophylaxis on the gut microbiota [64, 65]. After the first administration of prophylaxis, disappearance of quinolone-susceptible *Enterobacteriaceae* and emergence of FQ-resistant bacteria were observed. Even though levels of FQ-susceptible *Enterobacteriaceae* could recover during subsequent cycles of prophylaxis, detection rates of FQ-resistant bacteria significantly increased after repeated prophylaxis (8/35 samples versus 20/33 samples, *p* = 0.0028). Moreover, detection frequency seemed to increase with the number of prophylactic treatment cycles [64]. Other studies have shown that antibiotic exposure causes long-lasting disturbances of the gut microbiota, which may be a necessary step towards acquisition of multidrug-resistant colonization [66, 67].

There is growing concern regarding a possible effect of microbiota composition on oncogenesis and response to anti-cancer treatment. After two independent studies in mice demonstrated the dependency of cisplatin and cyclophosphamide efficacy on prevalence of distinct bacterial groups in the gut microbiota [68, 69], several clinical studies have confirmed that antibiotic exposure and/or gut microbiota composition may influence outcomes of human cancer patients as well [70–75]. Additional studies have highlighted the interconnection of antibiotic treatment and dysbiosis with the emergence of CDI, bloodstream infections and graft-versus-host disease (GvHD) in allogeneic stem cell recipients [76–80]. More recently, antibiotic use and dysbiosis have been linked to worse outcomes of viral infections, possibly as result of impaired T cell activation [81–83].

Further studies are needed to better understand the molecular mechanisms of the observed effects. There are currently no studies specifically reporting a potential effect of FQ prophylaxis on tumor response. However, it should be kept in mind that administration of antibiotic drugs may have considerable interaction with anti-cancer treatment. Any decision for antibiotic prophylaxis must be carefully balanced against the potential for resistance induction, immediate drug-related adverse events (e.g., rash, nausea, QT prolongation, and tendinopathy), and microbiota-mediated adverse events (e.g., CDI, antibiotic-associated diarrhea, GvHD, reduced effectiveness of anti-cancer treatment). It is imperative that future studies on antibiotic prophylaxis consider these new insights to fully evaluate the consequences of FQ exposure.

## Efficacy of prophylaxis

Antibacterial prophylaxis has been shown to be effective and safe in preventing febrile episodes and bacterial infections in neutropenic patients [5, 6, 13, 84–86]. A large meta-analysis conducted by Gafter-Gvili et al. included data from 13,579 patients out of 109 trials and compared prophylactic strategies (FQ, trimethoprim–sulfamethoxazole/TMP-SMX, other systemic drugs, non-absorbable drugs) with placebo or no intervention [13]. A significant reduction of infection-related and all-cause mortality could be shown in patients receiving antibacterial prophylaxis. Moreover, a significant decrease in rates of microbiologically documented infection and bacteremia could be observed. However, the meta-analyses included numerous historical studies from a different era of bacterial resistance and when proper empirical treatment strategies for FN were not universally established. Administration of antibacterial prophylaxis was generally associated with more side effects [13].

Following publications on the rapid and profound effect of antibiotics on selection of resistant bacteria and microbiota composition, it has become a matter of debate how a single drug prophylaxis can maintain effectiveness over a prolonged period of time, i.e., multiple chemotherapy cycles. Indeed, only one of the included studies [35] discriminated the outcome of overall and infection-related mortality between the first and each subsequent cycle while one additional study reported on outcomes during first and during all chemotherapy cycles [6]. It was therefore decided to separate evidence rating and recommendation for first and subsequent chemotherapy cycles.

Since the last version of this guideline [12], a comprehensive literature search by multiple researchers did not reveal new evidence from prospective, randomized clinical trials in adult cancer patients. The results of the recently published TEAMM trial indicate that prophylaxis with levofloxacin is significantly associated with a reduction in febrile episodes and death during the first 12 weeks of multiple myeloma (MM) therapy [87]. This led the authors to the conclusion that levofloxacin prophylaxis should become standard of care for newly diagnosed MM patients, although overall survival during the 1-year follow-up period of the trial was lower than in the placebo group. A major limitation of the TEAMM trial is that incidence of neutropenia was not reported for the patient cohorts, while administration of antibiotic prophylaxis in prolonged neutropenia is known to show a direct benefit for affected patients. A new meta-analysis evaluating the impact of antibiotic prophylaxis in multiple myeloma (MM) patients including the TEAMM trial indicated that antibiotic prophylaxis for the first 3 months following MM diagnosis decreases the incidence of infection, while this decrease did just reach the threshold of statistical significance. Moreover, no evidence for a decrease in mortality could be noted [88]. The modest benefit of antibacterial prophylaxis in MM patients has thus to be weighed carefully against the risk of resistance development, toxicity, and its impact on following therapies. Given the established quality of evidence, after thorough review of the identified literature, it was thus decided that the methodology of most new studies was insufficient to allow relevant new conclusions on prophylactic efficacy [55, 89–96].

However, the current studies underline that antibacterial prophylaxis continues to be effective in the current overall epidemiologic setting regarding predominant bacterial strains and resistances.

Based on the available evidence and under consideration of the defined risk groups, we recommend using antibacterial prophylaxis in high-risk patients receiving their first chemotherapy cycle if prevention of fever and infection is desired (A-I). This strategy may also be effective in reducing overall mortality, but ultimate evidence is lacking (B-II). Antibiotic prophylaxis in low-risk groups receiving their first chemotherapy cycle may be considered for prevention of fever and infection (B-I), but a reduction of mortality was not shown for this patient group (C-II). In this sense, low-risk patients with relevant individual risk factors should be considered as high-risk patients (Tables 2 and 3). Considering the increase of resistant bacteria and given lack of evidence, we downgraded our recommendation for antibiotic prophylaxis during subsequent chemotherapy cycles in high-risk (B-I) and low-risk patients (C-I) (recommendations summarized in Table 4).

**Table 4:** Indications for antibacterial prophylaxis

Clinical setting	Intention/recommendation	Intervention	SoR	QoE	References
High-risk patients receiving first chemotherapy cycle	Prevent fever and infections by using antibacterial prophylaxis^a^	Antibacterial prophylaxis	A	I	[5, 13, 66, 67, 70, 79, 97, 98]
High-risk patients receiving subsequent chemotherapy cycles			B	I	
Low-risk patients receiving first chemotherapy cycle			B	I	[6, 13, 66, 67, 70, 79, 97, 98]
Low-risk patients receiving subsequent chemotherapy cycles			C	I	
High-risk patients	Reduce mortality by using antibacterial prophylaxis		B	II	[5, 13, 84]
Low-risk patients			C	II	[6, 84, 98]
Patients receiving eculizumab, ravulizumab, or splenectomy or patients with functional asplenia without effective meningococcal vaccination	Prevent meningococcal disease	Penicillin V 250 mg b.i.d. or ciprofloxacin 500 mg q.d. until 4 weeks after immunization or documented protective titers	A	II_u_	[39, 40]

^a^The recommendation levels refer to the efficacy in achieving the specific endpoint and not on the overall recommendation of antibacterial prophylaxis. Prevention of fever and infections is a non-critical clinical goal and must be weighed against selective pressure and adverse drug effects (see sections “Recent warnings regarding fluoroquinolone safety” and “Novel considerations regarding microbiome and resistome”)

## Duration of prophylaxis administration

Data regarding timing of prophylaxis initiation and duration of administration are still scarce and comparative studies are warranted. In patients with high risk for development of FN and infection, prophylactic agents are mostly administered with the start of chemotherapy [5], while administration in low-risk patients is often initiated at the end of chemotherapy application [6]. We recommend starting antibacterial prophylaxis in high-risk patients with the start of the cytostatic regimen (B-II_u_). To reduce side effects and to prevent resistance development, antibacterial prophylaxis should be terminated with the end of neutropenia or with the initiation of therapeutic broad-spectrum antibiotics, as it has been protocol standard in the respective landmark trials [5, 6] (A-II_u_). In case of breakthrough infection during prophylactic administration of FQ, we recommend switching to another drug class for empiric therapy (A-III) (Table 5).

**Table 5:** Timing and duration of antibacterial prophylaxis

Clinical setting	Intention/recommendation	Intervention	SoR	QoE	References
Patients with indication for antibacterial prophylaxis at high risk for infection	Prevention of fever or infection	Start antibacterial prophylaxis with start of cytostatic drugs	B	II_u_	[5, 6, 13]
Patients with indication for antibacterial prophylaxis at low risk for infection		Start antibacterial prophylaxis 5–8 days after beginning chemotherapy	B	III	
Start of empirical broad-spectrum antibiotic treatment OR End of neutropenia	Reduce side effects, prevent resistance development	Termination of antibacterial prophylaxis	A	II_u_	
Patient with breakthrough infection receiving FQ prophylaxis	Treatment of infection	Use of FQ for empirical therapy	D	III	

## Drugs for antibacterial prophylaxis

Antibacterial prophylaxis in neutropenic patients is commonly performed with trimethoprim–sulfamethoxazole (TMP-SMX) in standard therapeutic dosing (800/160 mg every 12 h) or FQ. No significant differences regarding incidence of fever and infection as well as all-cause or infection-related mortality could be shown between both drugs [13, 99, 100]. The combination TMP-SMX is probably similarly effective as FQ for prevention of febrile neutropenia and death (B-II_t_). However, FQ prophylaxis is associated with better tolerability as well as lower bacterial resistance [13, 101]. Therefore, we recommend preferring FQs over TMP-SMX to avoid side effects (A-II). Compared to FQ, TMP-SMX has the advantage of providing additional prophylaxis against PcP [100]. Of note, we could not identify a study addressing the situation that a patient needs antibacterial and PcP prophylaxis at the same time. It remains unknown whether TMP-SMX three times per week in combination with FQ is better tolerated and/or more effective than TMP-SMX in therapeutic dose without FQ.

The FQ ciprofloxacin and levofloxacin have both been evaluated in different studies and are the first-choice agents for antibacterial prophylaxis compared to other FQs (A-II). Oral absorption of ciprofloxacin following chemotherapy has been described to decrease after cytostatic treatment [102].

Levofloxacin shows inferior efficacy against *Pseudomonas aeruginosa* than ciprofloxacin but has the advantage of being active against gram-positive bacteria. To date, other than ciprofloxacin, prophylactic administration of levofloxacin in patients with chemotherapy-associated neutropenia is not approved in Germany. Moreover, severe side effects including life-threatening hepatotoxicity led to an official warning regarding the off-label use of levofloxacin. Although not part of the major studies, it may be considered to use intravenous FQ formulations in patients with expected poor resorption due to severe diarrhea and/or inability to swallow the drug.

The broad-spectrum FQ moxifloxacin has been shown to prevent bacteremia in patients undergoing autologous HSCT and to shorten the length of febrile episodes [103]. However, it has no proven superiority to ciprofloxacin or levofloxacin prophylaxis, while it seems to be related with higher risk for CDI [104]. Furthermore, bacteremia due to *P. aeruginosa* could be observed during moxifloxacin prophylaxis [103].

It has been studied whether combining FQs with agents active against gram-positive bacteria prevents FN by reducing the incidence of gram-positive infections in neutropenic patients [35, 105]. A meta-analysis by Paul et al. showed no significant differences in all-cause mortality after addition of anti-gram-positive antibiotics, while bacterial superinfections due to gram-positive pathogens were less frequently detected [106]. Adverse events and nephrotoxicity were significantly higher when additional antibiotics were administered. Based on these findings, we do not recommend the addition of prophylactic antibiotics active against gram-positive bacteria in neutropenic patients (D-II) (Table 6).

**Table 6:** Drugs for antibacterial prophylaxis

Clinical setting	Intention/recommendation	Intervention	SoR	QoE	References
Neutropenic patients with indication for antibacterial prophylaxis^a^	Prevent FN or death	Use fluoroquinolone prophylaxis, if indicated	A	I	[13, 101, 107, 108]
	Prevent FN or death	Use therapeutic dose TMP-SMX instead of FQs, if indicated	B	II_t_	[13, 101, 107, 108]
	Prevent FN or death	Prefer selective gut decontamination vs. systemically active antibacterials	*		[109, 110]
	Reduce side effects of antibacterials	Prefer fluoroquinolone prophylaxis vs. TMP-SMX	A	II	[13, 101, 107, 108]
	Prevent FN or death	Ciprofloxacin and levofloxacin as FQs of choice for prophylaxis	A	II	[5, 13, 103–105]
	Prevent FN and reduce incidence of gram-positive infections	Combine fluoroquinolone with an agent active against gram-positive bacteria	D	II	[13, 35, 105, 106]
Neutropenic patients with indication for antibacterial prophylaxis and known colonization with multiresistant bacteria	Prevent FN or death	FQ prophylaxis if known colonization by FQ-resistant gram-negative bacteria	D	II_t, u_	[50, 51, 58, 79]

*No recommendation due to lack of randomized studies and conflicting data

^a^Note that these recommendations are made provided that a patient was determined to receive an antibacterial prophylaxis under consideration of Table 4. A high level of evidence/recommendation in this table does not provide recommendation to the indication of prophylaxis, but only on the choice of drug if an indication is given

## Recent warnings regarding fluoroquinolone safety

In recent years, a series of warnings regarding safety of FQs was released by several health authorities, including the German *Bundesinstitut für Arzneimittel und Medizinprodukte* and the US Food and Drug Administration. These warnings raise the issue of potentially disabling and permanent toxic effects on large blood vessels, muscles, tendons, joints, peripheral nerves, and the central nervous system [111–114]. Consequently, FQs should no longer be prescribed for non-severe and/or self-limiting infections (e.g., upper respiratory tract infections, urinary tract infection, acute exacerbation of chronic bronchitis, and COPD), traveler’s diarrhea, and chronic non-bacterial prostatitis. Special attention is required for patients at risk of toxicity, i.e., elderly patients, patients with impaired renal function, solid organ transplantation recipients, and patients receiving concomitant corticosteroids. Patients showing any signs of tendinitis or tendon rupture, myalgia, muscle weakness, arthralgia, neuropathy, confusion, or hallucinations. In addition, a list of preconditions increasing the risk for aortic aneurysms is provided in the warning letters.

The warning letters were discussed by the guideline group and it was agreed upon that the new evidence does not change the overall rating of the evidence or the recommendation. This has also been extensively argued by another guideline group [115]. The decision not to change the recommendation was based on three primary reasons. Firstly, in the setting of neutropenia, for no other drugs, sufficient evidence regarding efficacy and tolerability is available. While TMP-SMX has comparable efficacy, it showed a higher rate of treatment-emergent adverse events in comparative trials. Secondly, hematologic and oncologic patients are typically closely monitored by the treating physician and toxic side effects could probably be detected earlier, allowing timely termination of FQ treatment. And finally, the guideline group felt that the overall low rate of severe side effects of FQs, especially compared to the severity of the underlying disease of neutropenic patients and the high efficacy of FQs in preventing infections, does not outweigh the potential benefits of FQ use.

We advise physicians to inform patients about potential FQ side effects and discuss the risks and benefits of alternative strategies, i.e., standard-dose TMP-SMX (more acute side effects), an oral third-generation cephalosporin (lack of evidence), or no antibiotic prophylaxis (increased risk of fever and hospitalization).

## Selective digestive tract decontamination

The rationale behind decontamination of the intestinal or digestive tract is to eliminate most bacteria which can cause endogenous infection by translocation from the digestive system ideally by non-absorbable anti-infective agents only [44]. Selective digestive tract decontamination (often termed SDD or SD) aims at leaving the anaerobic flora intact and many authors of comparative studies have claimed a benefit for SDD 15–20 years ago [116–118]. However, most studies used absorbable antibiotics like cotrimoxazole or FQ and are thus difficult to distinguish from systemically active antibiotic prophylaxis. After a decade of no new data on SDD, two retrospective studies comparing non-absorbable antibiotics (colistin or rifaximin) with antibiotic prophylaxis using FQ in patients who received an allogeneic HSCT found comparable efficacy of SDD versus systemic antibiotics with regard to infections [109, 110]. The most recent study observed a survival benefit for patients who received non-absorbable as opposed to systemic antibiotic prophylaxis; however, it remains unclear how rifaximin would have compared to placebo or other systemically active antibiotic prophylaxis [110]. Of note, both studies do not define their regimen for PcP prophylaxis, which might add a low level of systemic antibiotic activity if given.

A concern regarding SDD is the possibility of inducing resistance. A recent meta-analysis of SDD in critically ill patients found no short-term increase of a small set of specific resistant organisms/resistance genes in studies investigating SDD [119], while other data indicate that the composition of the intestinal microbiota and the resistome is importantly affected by SDD [66, 120, 121]. It could be shown that application of SDD in intensive care patients has considerable effects on the gut microbiota, including overgrowth by *Enterococcus* spp. [121, 122].

In summary, because of the lack of well-designed prospective trials and the lack of data from patients other than allogeneic HSCT recipients, a recommendation regarding SDD cannot be made.

## Indication for *Pneumocystis jirovecii* prophylaxis

Infections caused by *Pneumocystis jirovecii* are a common complication in immunocompromised patients; the individual risk depends on the underlying disease and treatment regimen [123–126]. Trials on PcP in HIV-uninfected cancer patients are sparse, but several risk factors for development of PcP have been identified [127, 128].

Oncologic patients at significant risk for PcP are those treated for acute lymphoblastic leukemia, those treated with a combination of fludarabine, cyclophosphamide, and rituximab [129], and patients undergoing allogeneic HSCT [128, 130]. Moreover, long-term use of steroids (> 20 mg/day prednisone equivalent for 4 weeks) increases the risk of developing PcP [129, 131, 132]. Patients with hematologic malignancies appear to have higher mortality rates once they develop PcP [133]. The administration of chemoprophylaxis in patients with one of these three risk factors is thus strongly recommended to prevent PcP (A-I) and reduce mortality (A-II_r_).

Other hematologic and oncologic patients may occasionally develop PcP, such as lymphoma patients treated with R-CHOP14 or escalated BEACOPP protocol, patients treated with nucleoside analogs or long-term anti-CD20 antibody therapy, patients receiving high-dose steroids in addition to a radiotherapy for brain malignancies, and patients with CD4 count < 200 cells/μl [127, 134, 135]. There are no studies demonstrating benefit of PcP chemoprophylaxis in these patient groups, though it is likely that the number needed to treat is considerably higher compared to the high-risk groups. Such patients at moderate risk may or may not receive chemoprophylaxis to prevent PcP (C-III) and to reduce mortality (C-III).

New immune-modulating agents might increase the risk for PcP [136–138]. Patients receiving idelalisib are at high risk for developing PcP and prophylaxis must be administered concurrently (drug label) [139–141]. Initiation of chemoprophylaxis at the beginning of therapy is recommended by the manufacturer and should be maintained for 2–6 months after stopping therapy. Increased rates of PcP and other opportunistic infections have been observed in a phase II pilot of alemtuzumab without administration of prophylaxis [142]. Some authors recommend chemoprophylaxis during treatment with alemtuzumab with TMP-SMX or an equivalent agent [143, 144]. If radiotherapy is combined with temozolomide, chemoprophylaxis is recommended and administered in clinical studies [145–147]. Based on the documented high-risk situation, we strongly recommend chemoprophylaxis against PcP for patients receiving temozolomide (in combination with radiotherapy), idelalisib, or alemtuzumab (A-II_u,t_) (Tables 7 and 8).

**Table 7:** Risk factors and indications for *Pneumocystis jirovecii*

Significant risk	Intermediate risk	Special indications
• Acute lymphoblastic leukemia • Allogeneic stem cell transplantation • Long-term steroids with > 20 mg q.d. prednisone equivalent for > 4 weeks • Fludarabine + cyclophosphamide + rituximab	• R-CHOP14 or escalated BEACOPP • Nucleoside analogs • Brain irradiation with high-dose steroids • CD4 cell count < 200/μl	• Alemtuzumab • Idelalisib (drug label) • Brain irradiation + temozolomide

**Table 8:** Indications for *Pneumocystis jirovecii* pneumonia prophylaxis

Clinical setting	Intention/recommendation	Intervention	SoR	QoE	Reference
Patients at significant risk for developing PcP	Prevent PcP	Use chemoprophylaxis	A	I	[125–133, 148–151]
Patients at intermediate risk			C	III	[127, 134, 135]
Patients receiving idelalisib, alemtuzumab, or temozolomide (with radiation) treatment			A	II_u, t_	[142, 144, 152]
Patients at significant risk for developing PcP	Reduce mortality	Use chemoprophylaxis	A	II_r_	[127]
Patients at intermediate risk			C	III	[127]

## Choice of drugs and doses for PcP prophylaxis

Administration of PcP prophylaxis has been extensively studied in HIV-positive patients [153] and non-HIV patients [127, 133, 154]. Based on the available evidence, the most effective prophylactic agent is TMP-SMX, which also offers protection for toxoplasmosis [153], nocardiosis [155], and actinomycosis. For these reasons, TMP-SMX is the first-choice agent for PcP prophylaxis (A-II_t,r_). It is unknown whether the low doses of TMP-SMX used for PcP prophylaxis are also effective in preventing other infections. No relevant differences in efficacy of different dosing strategies for TMP-SMX, i.e., one single-strength tablet per day (80/400 mg) or one double-strength tablet (160/800 mg) daily or thrice a week, were shown in clinical studies [156, 157]. A study in HIV-infected patients observed a 43% lower risk of discontinuing TMP-SMX due to side effects if the double-strength tablet was given thrice a week instead of daily [153]. We recommend using any of these dosing regimens (B-II_t_).

In patients with intolerance or severe adverse events related to TMP-SMX administration, atovaquone, dapsone, or pentamidine (aerosolized) can be used as alternatives. Atovaquone has been studied in HIV-infected patients [158–160], while only few studies in non-HIV-infected immunocompromised patients exist [161–163]. The drug was highly effective, generally well tolerated and not associated with hematologic toxicity. We therefore recommend atovaquone as first alternative to TMP-SMX as PcP prophylaxis in patients with underlying hematologic or oncologic malignancies. Low-dose atovaquone has been described to be less effective in PcP prophylaxis [164]; thus, 1500 mg atovaquone should be administered once daily (off-label use) (A-II_t_). To increase bioavailability of atovaquone, it should be taken during a fatty meal [165, 166]. If hematologic toxicity is not a major concern, dapsone (100 mg/day) can be used (A-II_t_). Efficacy of daily dapsone was equivalent to the administration of TMP-SMX in comparative studies [167–169]. Testing for glucose-6-phosphat dehydrogenase deficiency is recommended prior to administration of dapsone. If prophylaxis of toxoplasmosis is indicated, dapsone should be combined with pyrimethamine. The risk of dapsone-induced methemoglobinemia must be carefully considered.

Aerosolized pentamidine is a popular option for PcP prophylaxis, considering the lack of hematologic toxicity or drug–drug interactions and the long dosing interval of 28 days. However, efficacy is probably lower compared to systemic prophylaxes. Pentamidine has no efficacy against toxoplasmosis and bacterial infections, which is of concern, especially after allogeneic HSCT [170–173]. Some authors have reported a mild to moderate decline in pulmonary function after long-term exposure [174–177], while others found no effect [178]. Pentamidine inhalation has been shown to cause considerable exposure and respiratory side effects in healthcare workers [179, 180], and adequate measures are recommended to protect staff. Our recommendation to use aerosolized pentamidine (300 mg once/month) is moderate (B-II_t_) (Table 9). There have been reports of successful use of intravenous pentamidine in prevention of PcP [187–189]. In the absence of comparative trials, no recommendation can be given for this regimen in favor of established ones.

**Table 9:** Drugs for ***Pneumocystis jirovecii*** pneumonia prophylaxis

Clinical setting	Intention/recommendation	Intervention	SoR	QoE	References
Patients with indication for PcP prophylaxis	Prevent PcP	Use TMP-SMX as first-choice agent	A	II_t, r_	[126–128, 133, 153, 154, 181, 182]
		Use one single-strength (80/400 mg) tablet daily **or** one double-strength tablet (160/800 mg) either daily **or** thrice a week	B	II_t_	[127, 153, 156, 157, 182–184]
Patients with intolerance or severe adverse effects due to TMP-SMX		Use **atovaquone** as second-choice drug - 1500 mg/day	A	II_t_	[37, 157, 160–162, 164]
		Use **dapsone** as second-choice drug - 100 mg/day	A	II_t_	[153, 160, 167]
		Use **pentamidine (aerosolized)** as second-choice drug - 300 mg once monthly	B	II_t_	[153, 159, 168, 170, 185, 186]

## Supplementary information

ESM 1 (DOCX 23 kb)

## Data Availability

Not applicable.

## References

[1] Kuderer NM, Dale DC, Crawford J, Cosler LE, Lyman GH (2006). Mortality, morbidity, and cost associated with febrile neutropenia in adult cancer patients. Cancer..

[2] Pagano L, Caira M, Rossi G, Tumbarello M, Fanci R, Garzia MG (2012). A prospective survey of febrile events in hematological malignancies. Ann Hematol.

[3] Cordonnier C, Cesaro S, Maschmeyer G, Einsele H, Donnelly JP, Alanio A (2016). Pneumocystis jirovecii pneumonia: still a concern in patients with haematological malignancies and stem cell transplant recipients. J Antimicrob Chemother.

[4] Heinz WJ, Buchheidt D, Christopeit M, von Lilienfeld-Toal M, Cornely OA, Einsele H et al (2017) Diagnosis and empirical treatment of fever of unknown origin (FUO) in adult neutropenic patients: guidelines of the Infectious Diseases Working Party (AGIHO) of the German Society of Hematology and Medical Oncology (DGHO). Ann Hematol 10.1007/s00277-017-3098-3 PMC5645428 28856437

[5] Bucaneve G, Micozzi A, Menichetti F, Martino P, Dionisi MS, Martinelli G (2005). Levofloxacin to prevent bacterial infection in patients with cancer and neutropenia. N Engl J Med.

[6] Cullen M, Steven N, Billingham L, Gaunt C, Hastings M, Simmonds P (2005). Antibacterial prophylaxis after chemotherapy for solid tumors and lymphomas. N Engl J Med.

[7] Hidalgo M, Mendiola C, Lopez-Vega JM, Castellano D, Mendez M, Batiste-Alenton E (1998). A multicenter randomized phase II trial of granulocyte-colony stimulating factor-supported, platinum-based chemotherapy with flexible midcycle cisplatin administration in patients with advanced ovarian carcinoma. PSAMOMA Cooperative Group. Spain Cancer.

[8] Heil G, Hoelzer D, Sanz MA, Lechner K, Liu Yin JA, Papa G (1997). A randomized, double-blind, placebo-controlled, phase III study of filgrastim in remission induction and consolidation therapy for adults with de novo acute myeloid leukemia. The International Acute Myeloid Leukemia Study Group. Blood..

[9] Truong J, Lee EK, Trudeau ME, Chan KK (2016). Interpreting febrile neutropenia rates from randomized, controlled trials for consideration of primary prophylaxis in the real world: a systematic review and meta-analysis. Ann Oncol.

[10] Liss BJ, Vehreschild JJ, Cornely OA, Hallek M, Fatkenheuer G, Wisplinghoff H (2012). Intestinal colonisation and blood stream infections due to vancomycin-resistant enterococci (VRE) and extended-spectrum beta-lactamase-producing Enterobacteriaceae (ESBLE) in patients with haematological and oncological malignancies. Infection..

[11] Vehreschild JJ, Bohme A, Cornely OA, Kahl C, Karthaus M, Kreuzer KA (2014). Prophylaxis of infectious complications with colony-stimulating factors in adult cancer patients undergoing chemotherapy-evidence-based guidelines from the Infectious Diseases Working Party AGIHO of the German Society for Haematology and Medical Oncology (DGHO). Ann Oncol.

[12] Neumann S, Krause SW, Maschmeyer G, Schiel X, von Lilienfeld-Toal M, Infectious Diseases Working P (2013). Primary prophylaxis of bacterial infections and Pneumocystis jirovecii pneumonia in patients with hematological malignancies and solid tumors: guidelines of the Infectious Diseases Working Party (AGIHO) of the German Society of Hematology and Oncology (DGHO). Ann Hematol.

[13] Gafter-Gvili A, Fraser A, Paul M, Vidal L, Lawrie TA, van de Wetering MD, et al. Antibiotic prophylaxis for bacterial infections in afebrile neutropenic patients following chemotherapy. Cochrane Database Syst Rev 2012;1:CD004386. 10.1002/14651858.CD004386.pub3 PMC4170789 22258955

[14] Cornely OA, Cuenca-Estrella M, Meis JF, Ullmann AJ (2014). European Society of Clinical Microbiology and Infectious Diseases (ESCMID) Fungal Infection Study Group (EFISG) and European Confederation of Medical Mycology (ECMM) 2013 joint guidelines on diagnosis and management of rare and emerging fungal diseases. Clin Microbiol Infect.

[15] Ullmann AJ, Schmidt-Hieber M, Bertz H, Heinz WJ, Kiehl M, Kruger W (2016). Infectious diseases in allogeneic haematopoietic stem cell transplantation: prevention and prophylaxis strategy guidelines 2016. Ann Hematol.

[16] Mellinghoff SC, Panse J, Alakel N, Behre G, Buchheidt D, Christopeit M et al (2017) Primary prophylaxis of invasive fungal infections in patients with haematological malignancies: 2017 update of the recommendations of the Infectious Diseases Working Party (AGIHO) of the German Society for Haematology and Medical Oncology (DGHO). Ann Hematol 10.1007/s00277-017-3196-2 PMC5754425 29218389

[17] Sandherr M, Hentrich M, von Lilienfeld-Toal M, Massenkeil G, Neumann S, Penack O (2015). Antiviral prophylaxis in patients with solid tumours and haematological malignancies--update of the Guidelines of the Infectious Diseases Working Party (AGIHO) of the German Society for Hematology and Medical Oncology (DGHO). Ann Hematol.

[18] Christopeit MS-H, M; Sprute, R.; Buchheidt, D.; Hentrich, M.; Karthaus, M.; Penack, O.; Ruhnke, M.; Weissinger, F.; Cornely, O.A.; Maschmeyer, G. Prophylaxis, diagnosis and therapy of infections in patients undergoing high-dose chemotherapy and autolo-gous hematopoietic stem cell transplantation. 2020 update of the recommendations of the Infectious Diseases Working Party (AGIHO) of the German Society of Hematology and Medical Oncology (DGHO). Submitted. 2020. 10.1007/s00277-020-04297-8 PMC7572248 33079221

[19] Slavin MA, Lingaratnam S, Mileshkin L, Booth DL, Cain MJ, Ritchie DS (2011). Use of antibacterial prophylaxis for patients with neutropenia. Australian Consensus Guidelines 2011 Steering Committee. Intern Med J.

[20] Taplitz RA, Kennedy EB, Bow EJ, Crews J, Gleason C, Hawley DK (2018). Antimicrobial prophylaxis for adult patients with cancer-related immunosuppression: ASCO and IDSA clinical practice guideline update. J Clin Oncol.

[21] Chao C, Page JH, Yang SJ, Rodriguez R, Huynh J, Chia VM (2014). History of chronic comorbidity and risk of chemotherapy-induced febrile neutropenia in cancer patients not receiving G-CSF prophylaxis. Ann Oncol.

[22] Lyman GH, Abella E, Pettengell R (2014). Risk factors for febrile neutropenia among patients with cancer receiving chemotherapy: a systematic review. Crit Rev Oncol Hematol.

[23] Skoetz N, Bohlius J, Engert A, Monsef I, Blank O, Vehreschild JJ. Prophylactic antibiotics or G(M)-CSF for the prevention of infections and improvement of survival in cancer patients receiving myelotoxic chemotherapy. Cochrane Database Syst Rev. 2015:CD007107. 10.1002/14651858.CD007107.pub3 PMC7389519 26687844

[24] Klastersky J, de Naurois J, Rolston K, Rapoport B, Maschmeyer G, Aapro M (2016). Management of febrile neutropaenia: ESMO Clinical Practice Guidelines. Ann Oncol.

[25] Flowers CR, Seidenfeld J, Bow EJ, Karten C, Gleason C, Hawley DK (2013). Antimicrobial prophylaxis and outpatient management of fever and neutropenia in adults treated for malignancy: American Society of Clinical Oncology clinical practice guideline. J Clin Oncol.

[26] Freifeld AG, Bow EJ, Sepkowitz KA, Boeckh MJ, Ito JI, Mullen CA (2011). Clinical practice guideline for the use of antimicrobial agents in neutropenic patients with cancer: 2010 update by the Infectious Diseases Society of America. Clin Infect Dis.

[27] Averbuch D, Orasch C, Cordonnier C, Livermore DM, Mikulska M, Viscoli C (2013). European guidelines for empirical antibacterial therapy for febrile neutropenic patients in the era of growing resistance: summary of the 2011 4th European Conference on Infections in Leukemia. Haematologica..

[28] Taplitz RA, Kennedy EB, Bow EJ, Crews J, Gleason C, Hawley DK, et al. (2018) Outpatient management of fever and neutropenia in adults treated for malignancy: American Society of Clinical Oncology and Infectious Diseases Society of America Clinical Practice Guideline Update. J Clin Oncol. JCO2017776211. 10.1200/JCO.2017.77.6211 29461916

[29] Baden LR, Swaminathan S, Angarone M, Blouin G, Camins BC, Casper C, et al. (2017) Prevention and treatment of cancer-related infections - NCCN Clinical Practice Guidelines in Oncology (NCCN Guidelines®) 10.6004/jnccn.2016.0093 27407129

[30] Lyman GH, Delgado DJ (2003). Risk and timing of hospitalization for febrile neutropenia in patients receiving CHOP, CHOP-R, or CNOP chemotherapy for intermediate-grade non-Hodgkin lymphoma. Cancer..

[31] Lyman GH, Morrison VA, Dale DC, Crawford J, Delgado DJ, Fridman M (2003). Risk of febrile neutropenia among patients with intermediate-grade non-Hodgkin’s lymphoma receiving CHOP chemotherapy. Leuk Lymphoma.

[32] Assi H, Murray J, Boyle L, Rayson D (2014). Incidence of febrile neutropenia in early stage breast cancer patients receiving adjuvant FEC-D treatment. Support Care Cancer.

[33] Borg C, Ray-Coquard I, Philip I, Clapisson G, Bendriss-Vermare N, Menetrier-Caux C (2004). CD4 lymphopenia as a risk factor for febrile neutropenia and early death after cytotoxic chemotherapy in adult patients with cancer. Cancer..

[34] Sasaki T, Takenaka Y, Hayashi T, Yamamoto M, Cho H, Fukusumi T (2015). Factors predicting severe infections during chemotherapy in head and neck cancer patients. Acta Otolaryngol.

[35] Tjan-Heijnen VC, PE P, A A, CH M, J B, J vM (2001). Reduction of chemotherapy-induced febrile leucopenia by prophylactic use of ciprofloxacin and roxithromycin in small-cell lung cancer patients: an EORTC double-blind placebo-controlled phase III study. Ann Oncol.

[36] Choi YW, Jeong SH, Ahn MS, Lee HW, Kang SY, Choi JH (2014). Patterns of neutropenia and risk factors for febrile neutropenia of diffuse large B-cell lymphoma patients treated with rituximab-CHOP. J Korean Med Sci.

[37] Chan A, Chen C, Chiang J, Tan SH, Ng R (2012). Incidence of febrile neutropenia among early-stage breast cancer patients receiving anthracycline-based chemotherapy. Support Care Cancer.

[38] Maschmeyer G, De Greef J, Mellinghoff SC, Nosari A, Thiebaut-Bertrand A, Bergeron A (2019). Infections associated with immunotherapeutic and molecular targeted agents in hematology and oncology. A position paper by the European Conference on Infections in Leukemia (ECIL). Leukemia..

[39] Benamu E, Montoya JG (2016). Infections associated with the use of eculizumab: recommendations for prevention and prophylaxis. Curr Opin Infect Dis.

[40] Bouts A, Monnens L, Davin JC, Struijk G, Spanjaard L (2011). Insufficient protection by Neisseria meningitidis vaccination alone during eculizumab therapy. Pediatr Nephrol.

[41] Mikulska M, Viscoli C, Orasch C, Livermore DM, Averbuch D, Cordonnier C (2014). Aetiology and resistance in bacteraemias among adult and paediatric haematology and cancer patients. J Inf Secur.

[42] Wisplinghoff H, Seifert H, Wenzel RP, Edmond MB (2003). Current trends in the epidemiology of nosocomial bloodstream infections in patients with hematological malignancies and solid neoplasms in hospitals in the United States. Clin Infect Dis.

[43] Gudiol C, Bodro M, Simonetti A, Tubau F, Gonzalez-Barca E, Cisnal M (2012). Changing aetiology, clinical features, antimicrobial resistance, and outcomes of bloodstream infection in neutropenic cancer patients. Clin Microbiol Infect.

[44] Marin M, Gudiol C, Ardanuy C, Garcia-Vidal C, Calvo M, Arnan M (2014). Bloodstream infections in neutropenic patients with cancer: differences between patients with haematological malignancies and solid tumours. J Inf Secur.

[45] Ramphal R (2004). Changes in the etiology of bacteremia in febrile neutropenic patients and the susceptibilities of the currently isolated pathogens. Clin Infect Dis.

[46] Conn JR, Catchpoole EM, Runnegar N, Mapp SJ, Markey KA (2017). Low rates of antibiotic resistance and infectious mortality in a cohort of high-risk hematology patients: a single center, retrospective analysis of blood stream infection. PLoS One.

[47] Cattaneo C, Quaresmini G, Casari S, Capucci MA, Micheletti M, Borlenghi E (2008). Recent changes in bacterial epidemiology and the emergence of fluoroquinolone-resistant Escherichia coli among patients with haematological malignancies: results of a prospective study on 823 patients at a single institution. J Antimicrob Chemother.

[48] Trecarichi EM, Pagano L, Candoni A, Pastore D, Cattaneo C, Fanci R (2015). Current epidemiology and antimicrobial resistance data for bacterial bloodstream infections in patients with hematologic malignancies: an Italian multicentre prospective survey. Clin Microbiol Infect.

[49] Weisser M, Theilacker C, Tschudin Sutter S, Babikir R, Bertz H, Gotting T et al (2017) Secular trends of bloodstream infections during neutropenia in 15181 haematopoietic stem cell transplants: 13-year results from a European multicentre surveillance study (ONKO-KISS). Clin Microbiol Infect 10.1016/j.cmi.2017.03.020 28366613

[50] Biehl LM, Schmidt-Hieber M, Liss B, Cornely OA, Vehreschild MJ (2016). Colonization and infection with extended spectrum beta-lactamase producing Enterobacteriaceae in high-risk patients - review of the literature from a clinical perspective. Crit Rev Microbiol.

[51] Vehreschild MJ, Hamprecht A, Peterson L, Schubert S, Hantschel M, Peter S (2014). A multicentre cohort study on colonization and infection with ESBL-producing Enterobacteriaceae in high-risk patients with haematological malignancies. J Antimicrob Chemother.

[52] De Rosa FG, Motta I, Audisio E, Frairia C, Busca A, Di Perri G (2013). Epidemiology of bloodstream infections in patients with acute myeloid leukemia undergoing levofloxacin prophylaxis. BMC Infect Dis.

[53] Vehreschild MJ, Weitershagen D, Biehl LM, Tacke D, Waldschmidt D, Tox U (2014). Clostridium difficile infection in patients with acute myelogenous leukemia and in patients undergoing allogeneic stem cell transplantation: epidemiology and risk factor analysis. Biol Blood Marrow Transplant.

[54] Kern WV, Weber S, Dettenkofer M, Kaier K, Bertz H, Behnke M (2018). Impact of fluoroquinolone prophylaxis during neutropenia on bloodstream infection: data from a surveillance program in 8755 patients receiving high-dose chemotherapy for haematologic malignancies between 2009 and 2014. J Inf Secur.

[55] Verlinden A, Jansens H, Goossens H, van de Velde AL, Schroyens WA, Berneman ZN (2014). Clinical and microbiological impact of discontinuation of fluoroquinolone prophylaxis in patients with prolonged profound neutropenia. Eur J Haematol.

[56] See I, Freifeld AG, Magill SS (2016). Causative organisms and associated antimicrobial resistance in healthcare-associated, central line-associated bloodstream infections from oncology settings, 2009-2012. Clin Infect Dis.

[57] Satlin MJ, Cohen N, Ma KC, Gedrimaite Z, Soave R, Askin G (2016). Bacteremia due to carbapenem-resistant Enterobacteriaceae in neutropenic patients with hematologic malignancies. J Inf Secur.

[58] Kantele A, Mero S, Kirveskari J, Laaveri T (2017) Fluoroquinolone antibiotic users select fluoroquinolone-resistant ESBL-producing Enterobacteriaceae (ESBL-PE) - data of prospective traveller study. Travel Med Infect Dis 10.1016/j.tmaid.2017.01.003 28153711

[59] Hakki M, Humphries RM, Hemarajata P, Tallman GB, Shields RK, Mettus RT (2019). Fluoroquinolone prophylaxis selects for meropenem-nonsusceptible Pseudomonas aeruginosa in patients with hematologic malignancies and hematopoietic cell transplant recipients. Clin Infect Dis.

[60] Saito T, Yoshioka S, Iinuma Y, Takakura S, Fujihara N, Ichinohe T (2008). Effects on spectrum and susceptibility patterns of isolates causing bloodstream infection by restriction of fluoroquinolone prophylaxis in a hematology-oncology unit. Eur J Clin Microbiol Infect Dis.

[61] Chong Y, Yakushiji H, Ito Y, Kamimura T (2011). Clinical impact of fluoroquinolone prophylaxis in neutropenic patients with hematological malignancies. Int J Infect Dis.

[62] Miles-Jay A, Butler-Wu S, Rowhani-Rahbar A, Pergam SA (2015). Incidence rate of fluoroquinolone-resistant gram-negative rod bacteremia among allogeneic hematopoietic cell transplantation patients during an era of levofloxacin prophylaxis. Biol Blood Marrow Transplant.

[63] Laukens D, Brinkman BM, Raes J, De Vos M, Vandenabeele P (2016). Heterogeneity of the gut microbiome in mice: guidelines for optimizing experimental design. FEMS Microbiol Rev.

[64] Chong Y, Shimoda S, Miyake N, Aoki T, Ito Y, Kamimura T (2017). Incomplete recovery of the fecal flora of hematological patients with neutropenia and repeated fluoroquinolone prophylaxis. Infect Drug Resist.

[65] Chong Y, Shimoda S, Yakushiji H, Ito Y, Aoki T, Miyamoto T (2014). Clinical impact of fluoroquinolone-resistant Escherichia coli in the fecal flora of hematological patients with neutropenia and levofloxacin prophylaxis. PLoS One.

[66] Buelow E, Gonzalez TB, Versluis D, Oostdijk EA, Ogilvie LA, van Mourik MS (2014). Effects of selective digestive decontamination (SDD) on the gut resistome. J Antimicrob Chemother.

[67] Isaac S, Scher JU, Djukovic A, Jimenez N, Littman DR, Abramson SB (2017). Short- and long-term effects of oral vancomycin on the human intestinal microbiota. J Antimicrob Chemother.

[68] Iida N, Dzutsev A, Stewart CA, Smith L, Bouladoux N, Weingarten RA (2013). Commensal bacteria control cancer response to therapy by modulating the tumor microenvironment. Science..

[69] Viaud S, Saccheri F, Mignot G, Yamazaki T, Daillere R, Hannani D (2013). The intestinal microbiota modulates the anticancer immune effects of cyclophosphamide. Science..

[70] Pflug N, Kluth S, Vehreschild JJ, Bahlo J, Tacke D, Biehl L (2016). Efficacy of antineoplastic treatment is associated with the use of antibiotics that modulate intestinal microbiota. Oncoimmunology..

[71] Peled JU, Devlin SM, Staffas A, Lumish M, Khanin R, Littmann ER (2017). Intestinal microbiota and relapse after hematopoietic-cell transplantation. J Clin Oncol.

[72] Weber D, Jenq RR, Peled JU, Taur Y, Hiergeist A, Koestler J (2017). Microbiota disruption induced by early use of broad-spectrum antibiotics is an independent risk factor of outcome after allogeneic stem cell transplantation. Biol Blood Marrow Transplant.

[73] Gopalakrishnan V, Spencer CN, Nezi L, Reuben A, Andrews MC, Karpinets TV (2018). Gut microbiome modulates response to anti-PD-1 immunotherapy in melanoma patients. Science..

[74] Elkrief A, El Raichani L, Richard C, Messaoudene M, Belkaid W, Malo J (2019). Antibiotics are associated with decreased progression-free survival of advanced melanoma patients treated with immune checkpoint inhibitors. Oncoimmunology..

[75] Pinato DJ, Howlett S, Ottaviani D, Urus H, Patel A, Mineo T (2019). Association of prior antibiotic treatment with survival and response to immune checkpoint inhibitor therapy in patients with cancer. JAMA Oncology.

[76] Lee YJ, Arguello ES, Jenq RR, Littmann E, Kim GJ, Miller LC (2017). Protective factors in the intestinal microbiome against clostridium difficile infection in recipients of allogeneic hematopoietic stem cell transplantation. J Infect Dis.

[77] Harris B, Morjaria SM, Littmann ER, Geyer AI, Stover DE, Barker JN (2016). Gut microbiota predict pulmonary infiltrates after allogeneic hematopoietic cell transplantation. Am J Respir Crit Care Med.

[78] Taur Y, Jenq RR, Perales MA, Littmann ER, Morjaria S, Ling L (2014). The effects of intestinal tract bacterial diversity on mortality following allogeneic hematopoietic stem cell transplantation. Blood..

[79] Taur Y, Xavier JB, Lipuma L, Ubeda C, Goldberg J, Gobourne A (2012). Intestinal domination and the risk of bacteremia in patients undergoing allogeneic hematopoietic stem cell transplantation. Clin Infect Dis.

[80] Routy B, Letendre C, Enot D, Chenard-Poirier M, Mehraj V, Seguin NC (2017). The influence of gut-decontamination prophylactic antibiotics on acute graft-versus-host disease and survival following allogeneic hematopoietic stem cell transplantation. Oncoimmunology..

[81] Thackray LB, Handley SA, Gorman MJ, Poddar S, Bagadia P, Briseno CG (2018). Oral antibiotic treatment of mice exacerbates the disease severity of multiple flavivirus infections. Cell Rep.

[82] Haak BW, Littmann ER, Chaubard JL, Pickard AJ, Fontana E, Adhi F (2018). Impact of gut colonization with butyrate-producing microbiota on respiratory viral infection following allo-HCT. Blood..

[83] Ogimi C, Krantz EM, Golob JL, Waghmare A, Liu C, Leisenring WM (2018). Antibiotic exposure prior to respiratory viral infection is associated with progression to lower respiratory tract disease in allogeneic hematopoietic cell transplant recipients. Biol Blood Marrow Transplant.

[84] Imran H, Tleyjeh IM, Arndt CA, Baddour LM, Erwin PJ, Tsigrelis C (2008). Fluoroquinolone prophylaxis in patients with neutropenia: a meta-analysis of randomized placebo-controlled trials. Eur J Clin Microbiol Infect Dis.

[85] Owattanapanich W, Chayakulkeeree M (2019). Efficacy of levofloxacin as an antibacterial prophylaxis for acute leukemia patients receiving intensive chemotherapy: a systematic review and meta-analysis. Hematology (Amsterdam, Netherlands).

[86] Egan G, Robinson PD, Martinez JPD, Alexander S, Ammann RA, Dupuis LL (2019). Efficacy of antibiotic prophylaxis in patients with cancer and hematopoietic stem cell transplantation recipients: a systematic review of randomized trials. Cancer Med.

[87] Drayson MT, Bowcock S, Planche T, Iqbal G, Pratt G, Yong K (2019). Levofloxacin prophylaxis in patients with newly diagnosed myeloma (TEAMM): a multicentre, double-blind, placebo-controlled, randomised, phase 3 trial. Lancet Oncol.

[88] Mohyuddin GR, Aziz M, McClune B, Abdallah AO, Qazilbash M (2020). Antibiotic prophylaxis for patients with newly diagnosed multiple myeloma: Systematic review and meta-analysis. Eur J Haematol.

[89] Yeshurun M, Vaxman I, Shargian L, Yahav D, Bishara J, Pasvolsky O et al (2017) Antibacterial prophylaxis with ciprofloxacin for patients with multiple myeloma and lymphoma undergoing autologous haematopoietic cell transplantation: a quasi-experimental single-centre before-after study. Clin Microbiol Infect 10.1016/j.cmi.2017.11.019 29208561

[90] Yemm KE, Barreto JN, Mara KC, Dierkhising RA, Gangat N, Tosh PK (2018). A comparison of levofloxacin and oral third-generation cephalosporins as antibacterial prophylaxis in acute leukaemia patients during chemotherapy-induced neutropenia. J Antimicrob Chemother.

[91] Modi D, Jang H, Kim S, Surapaneni M, Sankar K, Deol A (2017). Fluoroquinolone prophylaxis in autologous hematopoietic stem cell transplant recipients. Support Care Cancer.

[92] Pohlen M, Marx J, Mellmann A, Becker K, Mesters RM, Mikesch JH (2016). Ciprofloxacin versus colistin prophylaxis during neutropenia in acute myeloid leukemia: two parallel patient cohorts treated in a single center. Haematologica..

[93] Satlin MJ, Vardhana S, Soave R, Shore TB, Mark TM, Jacobs SE (2015). Impact of prophylactic levofloxacin on rates of bloodstream infection and fever in neutropenic patients with multiple myeloma undergoing autologous hematopoietic stem cell transplantation. Biol Blood Marrow Transplant.

[94] Jung SH, Kang SJ, Jang HC, Ahn JS, Yang DH, Lee SS (2014). Effect of levofloxacin prophylaxis for prevention of severe infections in multiple myeloma patients receiving bortezomib-containing regimens. Int J Hematol.

[95] Lee SSF, Fulford AE, Quinn MA, Seabrook J, Rajakumar I (2018). Levofloxacin for febrile neutropenia prophylaxis in acute myeloid leukemia patients associated with reduction in hospital admissions. Support Care Cancer.

[96] Fernandes R, Mazzarello S, Stober C, Ibrahim MFK, Dudani S, Perdrizet K (2018). Primary febrile neutropenia prophylaxis for patients who receive FEC-D chemotherapy for breast cancer: a systematic review. J Glob Oncol.

[97] Gafter-Gvili A, Fraser A, Paul M, Leibovici L (2005). Meta-analysis: antibiotic prophylaxis reduces mortality in neutropenic patients. Ann Intern Med.

[98] Leibovici L, Paul M, Cullen M, Bucaneve G, Gafter-Gvili A, Fraser A (2006). Antibiotic prophylaxis in neutropenic patients: new evidence, practical decisions. Cancer..

[99] Bow EJ, Rayner E, Louie TJ (1988). Comparison of norfloxacin with cotrimoxazole for infection prophylaxis in acute leukemia. Am J Med.

[100] Mayer K, Hahn-Ast C, Muckter S, Schmitz A, Krause S, Felder L (2015). Comparison of antibiotic prophylaxis with cotrimoxazole/colistin (COT/COL) versus ciprofloxacin (CIP) in patients with acute myeloid leukemia. Support Care Cancer.

[101] Dekker AW (1987). Infection prophylaxis in acute leukemia: a comparison of ciprofloxacin with trimethoprim-sulfamethoxazole and colistin. Ann Intern Med.

[102] Johnson EJ, MacGowan AP, Potter MN, Stockley RK, White LO, Slade RR (1990). Reduced absorption of oral ciprofloxacin after chemotherapy for haematological malignancy. J Antimicrob Chemother.

[103] Vehreschild JJ, Moritz G, Vehreschild MJ, Arenz D, Mahne M, Bredenfeld H (2012). Efficacy and safety of moxifloxacin as antibacterial prophylaxis for patients receiving autologous haematopoietic stem cell transplantation: a randomised trial. Int J Antimicrob Agents.

[104] von Baum H, Sigge A, Bommer M, Kern WV, Marre R, Dohner H (2006). Moxifloxacin prophylaxis in neutropenic patients. J Antimicrob Chemother.

[105] Cruciani M, Malena M, Bosco O, Nardi S, Serpelloni G, Mengoli C (2003). Reappraisal with meta-analysis of the addition of Gram-positive prophylaxis to fluoroquinolone in neutropenic patients. J Clin Oncol.

[106] Paul M, Borok S, Fraser A, Vidal L, Leibovici L (2005). Empirical antibiotics against Gram-positive infections for febrile neutropenia: systematic review and meta-analysis of randomized controlled trials. J Antimicrob Chemother.

[107] Bow EJ, Rayner E, Louie TJ (1988). Comparison of norfloxacin with cotrimoxazole for infection prophylaxis in acute leukemia. The trade-off for reduced gram-negative sepsis. Am J Med.

[108] Donnelly JP, Maschmeyer G, Daenen S (1992). Selective oral antimicrobial prophylaxis for the prevention of infection in acute leukaemia-ciprofloxacin versus co-trimoxazole plus colistin. The EORTC-Gnotobiotic Project Group. Eur J Cancer (Oxford, England : 1990).

[109] Koh S, Yamada K, Nishimoto M, Hayashi Y, Koh H, Nakashima Y (2015). Effectiveness of antibacterial prophylaxis with non-absorbable polymyxin B compared to levofloxacin after allogeneic hematopoietic stem cell transplantation. Transpl Infect Dis.

[110] Weber D, Oefner PJ, Dettmer K, Hiergeist A, Koestler J, Gessner A (2016). Rifaximin preserves intestinal microbiota balance in patients undergoing allogeneic stem cell transplantation. Bone Marrow Transplant.

[111] FDA Drug Safety Communication: FDA advises restricting fluoroquinolone antibiotic use for certain uncomplicated infections; warns about disabling side effects that can occur together. 2016.

[112] FDA warns about increased risk of ruptures or tears in the aorta blood vessel with fluoroquinolone antibiotics in certain patients. US Food and Drug Administration; 2018.

[113] Rote-Hand-Brief zu systemisch und inhalativ angewendeten Fluorchinolonen: Risiko für Aortenaneurysmen und Aortendissektionen German Bundesinstitut für Arzneimittel und Medizinprodukte 2018.

[114] Rote-Hand-Brief zu Fluorchinolon-Antibiotika: Schwerwiegende und anhaltende, die Lebensqualität beeinträchtigende und möglicherweise irreversible Nebenwirkungen. German Bundesinstitut für Arzneimittel und Medizinprodukte; 2019.

[115] Mikulska M, Averbuch D, Tissot F, Cordonnier C, Akova M, Calandra T (2018). Fluoroquinolone prophylaxis in haematological cancer patients with neutropenia: ECIL critical appraisal of previous guidelines. J Inf Secur.

[116] Beelen DW, Elmaagacli A, Müller KD, Hirche H, Schaefer UW (1999). Influence of intestinal bacterial decontamination using metronidazole and ciprofloxacin or ciprofloxacin alone on the development of acute graft-versus-host disease after marrow transplantation in patients with hematologic malignancies: final results and long-term follow-up of an open-label prospective randomized trial. Blood..

[117] Watson JG, Jameson B, Powles RL, McElwain TJ, Lawson DN, Judson I (1982). Co-trimoxazole versus non-absorbable antibiotics in acute leukaemia. Lancet.

[118] Bow EJ, Rayner E, Scott BA, Louie TJ (1987). Selective gut decontamination with nalidixic acid or trimethoprim-sulfamethoxazole for infection prophylaxis in neutropenic cancer patients: relationship of efficacy to antimicrobial spectrum and timing of administration. Antimicrob Agents Chemother.

[119] Daneman N, Sarwar S, Fowler RA, Cuthbertson BH (2013). Effect of selective decontamination on antimicrobial resistance in intensive care units: a systematic review and meta-analysis. Lancet Infect Dis.

[120] Benus RF, Harmsen HJ, Welling GW, Spanjersberg R, Zijlstra JG, Degener JE (2010). Impact of digestive and oropharyngeal decontamination on the intestinal microbiota in ICU patients. Intensive Care Med.

[121] Buelow E, Bello Gonzalez TDJ, Fuentes S, de Steenhuijsen Piters WAA, Lahti L, Bayjanov JR (2017). Comparative gut microbiota and resistome profiling of intensive care patients receiving selective digestive tract decontamination and healthy subjects. Microbiome..

[122] Bello Gonzalez TDJ, Pham P, Top J, Willems RJL, van Schaik W, van Passel MWJ (2017). Characterization of Enterococcus isolates colonizing the intestinal tract of intensive care unit patients receiving selective digestive decontamination. Front Microbiol.

[123] Schoovaerts K, Dirix L, Rutten A, Van Schaeren J, Van Herendael B, Van Grieken S et al (2017) Pneumocystis jiroveci pneumonia (PJP) in non-HIV immunocompromised individuals. Acta Clin Belg:1–4 10.1080/17843286.2017.1305136 28346081

[124] Walzer PD, Perl DP, Krogstad DJ, Rawson PG, Schultz MG (1974). Pneumocystis carinii pneumonia in the United States. Epidemiologic, diagnostic, and clinical features. Ann Intern Med.

[125] Hughes WT, Feldman S, Aur RJ, Verzosa MS, Hustu HO, Simone JV (1975). Intensity of immunosuppressive therapy and the incidence of Pneumocystis carinii pneumonitis. Cancer..

[126] Sepkowitz KA (2002). Opportunistic infections in patients with and patients without Acquired immunodeficiency syndrome. Clin Infect Dis.

[127] Stern A, Green H, Paul M, Vidal L, Leibovici L (2014) Prophylaxis for Pneumocystis pneumonia (PCP) in non-HIV immunocompromised patients. Cochrane Database Syst Rev. CD005590. 10.1002/14651858.CD005590.pub2 17636808

[128] Hughes WT, Price RA, Kim H-K, Coburn TP, Grigsby D, Feldman S (1973). Pneumocystis carinii pneumonitis in children with malignancies. J Pediatr.

[129] Martin-Garrido I, Carmona EM, Specks U, Limper AH (2013). Pneumocystis pneumonia in patients treated with rituximab. Chest..

[130] De Castro N, Neuville S, Sarfati C, Ribaud P, Derouin F, Gluckman E (2005). Occurrence of Pneumocystis jiroveci pneumonia after allogeneic stem cell transplantation: a 6-year retrospective study. Bone Marrow Transplant.

[131] Mansharamani NG, Garland R, Delaney D, Koziel H (2000). Management and outcome patterns for adult Pneumocystis carinii pneumonia, 1985 to 1995. Chest..

[132] Maeda T, Babazono A, Nishi T, Matsuda S, Fushimi K, Fujimori K (2015). Quantification of the effect of chemotherapy and steroids on risk of Pneumocystis jiroveci among hospitalized patients with adult T-cell leukaemia. Br J Haematol.

[133] Sepkowitz KA (1993). Pneumocystis carinii pneumonia in patients without AIDS. Clin Infect Dis.

[134] Huang YC, Liu CJ, Liu CY, Pai JT, Hong YC, Teng HW (2011). Low absolute lymphocyte count and addition of rituximab confer high risk for interstitial pneumonia in patients with diffuse large B-cell lymphoma. Ann Hematol.

[135] Kolstad A, Holte H, Fosså A, Lauritzsen GF, Gaustad P, Torfoss D (2007). Pneumocystis jirovecii pneumonia in B-cell lymphoma patients treated with the rituximab-CHOEP-14 regimen. Haematologica..

[136] Mikulska M, Lanini S, Gudiol C, Drgona L, Ippolito G, Fernandez-Ruiz M et al (2018) ESCMID Study Group for Infections in Compromised Hosts (ESGICH) Consensus Document on the safety of targeted and biological therapies: an infectious diseases perspective (agents targeting lymphoid cells surface antigens [I]: CD19, CD20 and CD52). Clin Microbiol Infect 10.1016/j.cmi.2018.02.003 29447988

[137] Baddley JW, Cantini F, Goletti D, Gomez-Reino JJ, Mylonakis E, San-Juan R et al (2018) ESCMID Study Group for Infections in Compromised Hosts (ESGICH) Consensus Document on the safety of targeted and biological therapies: an infectious diseases perspective (Soluble immune effector molecules [I]: anti-tumour necrosis factor-alpha agents). Clin Microbiol Infect 10.1016/j.cmi.2017.12.025 29459143

[138] Fernandez-Ruiz M, Meije Y, Manuel O, Akan H, Carratala J, Aguado JM (2018). ESCMID Study Group for Infections in Compromised Hosts (ESGICH) Consensus Document on the safety of targeted and biological therapies: an infectious diseases perspective (introduction). Clin Microbiol Infect.

[139] Zelenetz AD, Barrientos JC, Brown JR, Coiffier B, Delgado J, Egyed M (2017). Idelalisib or placebo in combination with bendamustine and rituximab in patients with relapsed or refractory chronic lymphocytic leukaemia: interim results from a phase 3, randomised, double-blind, placebo-controlled trial. Lancet Oncol.

[140] Salles G, Schuster SJ, de Vos S, Wagner-Johnston ND, Viardot A, Blum KA (2017). Efficacy and safety of idelalisib in patients with relapsed, rituximab- and alkylating agent-refractory follicular lymphoma: a subgroup analysis of a phase 2 study. Haematologica..

[141] Reinwald M, Silva JT, Mueller NJ, Fortun J, Garzoni C, de Fijter JW et al (2018) ESCMID Study Group for Infections in Compromised Hosts (ESGICH) Consensus Document on the safety of targeted and biological therapies: an infectious diseases perspective (intracellular signaling pathways: tyrosine kinase and mTOR inhibitors). Clin Microbiol Infect 10.1016/j.cmi.2018.02.009 29454849

[142] Rai KR, Freter CE, Mercier RJ, Cooper MR, Mitchell BS, Stadtmauer EA (2002). Alemtuzumab in previously treated chronic lymphocytic leukemia patients who also had received fludarabine. J Clin Oncol.

[143] Keating M, Coutré S, Rai K, Österborg A, Faderl S, Kennedy B (2004). Management guidelines for use of alemtuzumab in B-cell chronic lymphocytic leukemia. Clinical Lymphoma.

[144] Martin SI, Marty FM, Fiumara K, Treon SP, Gribben JG, Baden LR (2006). Infectious complications associated with alemtuzumab use for lymphoproliferative disorders. Clin Infect Dis.

[145] Grossman SA, Ye X, Lesser G, Sloan A, Carraway H, Desideri S (2011). Immunosuppression in patients with high-grade gliomas treated with radiation and temozolomide. Clin Cancer Res.

[146] Neuwelt AJ, Nguyen TM, Fu R, Bubalo J, Tyson RM, Lacy C (2014). Incidence of Pneumocystis jirovecii pneumonia after temozolomide for CNS malignancies without prophylaxis. CNS Oncol.

[147] Stupp R, Mason WP, van den Bent MJ, Weller M, Fisher B, Taphoorn MJ (2005). Radiotherapy plus concomitant and adjuvant temozolomide for glioblastoma. N Engl J Med.

[148] Siegel SE, Nesbit ME, Baehner R, Sather H, Hammond GD (1980). Pneumonia during therapy for childhood acute lymphoblastic leukemia. Am J Dis Child (1960).

[149] Kulke MH, Vance EA (1997). Pneumocystis carinii pneumonia in patients receiving chemotherapy for breast cancer. Clin Infect Dis.

[150] Obeid KM, Aguilar J, Szpunar S, Sharma M, del Busto R, Al-Katib A (2012). Risk factors for Pneumocystis jirovecii pneumonia in patients with lymphoproliferative disorders. Clinical lymphoma, myeloma & leukemia.

[151] Waks AG, Tolaney SM, Galar A, Arnaout A, Porter JB, Marty FM (2015). Pneumocystis jiroveci pneumonia (PCP) in patients receiving neoadjuvant and adjuvant anthracycline-based chemotherapy for breast cancer: incidence and risk factors. Breast Cancer Res Treat.

[152] Elter T, Vehreschild JJ, Gribben J, Cornely OA, Engert A, Hallek M (2009). Management of infections in patients with chronic lymphocytic leukemia treated with alemtuzumab. Ann Hematol.

[153] Ioannidis JP, Cappelleri JC, Skolnik PR, Lau J, Sacks HS (1996). A meta-analysis of the relative efficacy and toxicity of Pneumocystis carinii prophylactic regimens. Arch Intern Med.

[154] Pagano L, Fianchi L, Mele L, Girmenia C, Offidani M, Ricci P (2002). Pneumocystis cariniipneumonia in patients with malignant haematological diseases: 10 years’ experience of infection in GIMEMA centres. Br J Haematol.

[155] Molina A, Winston DJ, Pan D, Schiller GJ (2018) Increased incidence of nocardial infections in an era of atovaquone prophylaxis in allogeneic hematopoietic stem cell transplant recipients. Biol Blood Marrow Transplant 10.1016/j.bbmt.2018.03.010 29555315

[156] Di Cocco P, Orlando G, Bonanni L, D'Angelo M, Clemente K, Greco S (2009). A systematic review of two different trimetoprim-sulfamethoxazole regimens used to prevent Pneumocystis jirovecii and no prophylaxis at all in transplant recipients: appraising the evidence. Transplant Proc.

[157] El-Sadr WM, Luskin-Hawk R, Yurik TM, Walker J, Abrams D, John SL (1999). A randomized trial of daily and thrice-weekly trimethoprim-sulfamethoxazole for the prevention of Pneumocystis carinii pneumonia in human immunodeficiency virus-infected persons. Terry Beirn Community Programs for Clinical Research on AIDS (CPCRA). Clin Infect Dis.

[158] Hughes WT, Dankner WM, Yogev R, Huang S, Paul ME, Flores MA (2005). Comparison of atovaquone and azithromycin with trimethoprim-sulfamethoxazole for the prevention of serious bacterial infections in children with HIV infection. Clin Infect Dis.

[159] Chan C, Montaner J, Lefebvre E-A, Morey G, Dohn M, McIvor RA (1999). Atovaquone suspension compared with aerosolized pentamidine for prevention of Pneumocystis carinii pneumonia in human immunodeficiency virus-infected subjects intolerant of trimethoprim or sulfonamides. J Infect Dis.

[160] El-Sadr WM, Murphy RL, Yurik TM, Luskin-Hawk R, Cheung TW, Balfour HH (1998). Atovaquone compared with dapsone for the prevention of Pneumocystis carinii pneumonia in patients with HIV infection who cannot tolerate trimethoprim, sulfonamides, or both. Community Program for Clinical Research on AIDS and the AIDS Clinical Trials Group. N Engl J Med.

[161] Colby C, McAfee S, Sackstein R, Finkelstein D, Fishman J, Spitzer T (1999). A prospective randomized trial comparing the toxicity and safety of atovaquone with trimethoprim/sulfamethoxazole as Pneumocystis carinii pneumonia prophylaxis following autologous peripheral blood stem cell transplantation. Bone Marrow Transplant.

[162] Gabardi S, Millen P, Hurwitz S, Martin S, Roberts K, Chandraker A (2012). Atovaquone versus trimethoprim-sulfamethoxazole as Pneumocystis jirovecii pneumonia prophylaxis following renal transplantation. Clin Transpl.

[163] Mendorf A, Klyuchnikov E, Langebrake C, Rohde H, Ayuk F, Regier M (2015). Atovaquone for prophylaxis of toxoplasmosis after allogeneic hematopoietic stem cell transplantation. Acta Haematol.

[164] Rodriguez M, Sifri CD, Fishman JA (2004). Failure of low-dose atovaquone prophylaxis against Pneumocystis jiroveci infection in transplant recipients. Clin Infect Dis.

[165] Freeman CD, Klutman NE, Lamp KC, Dall LH, Strayer AH (1998). Relative bioavailability of atovaquone suspension when administered with an enteral nutrition supplement. Ann Pharmacother.

[166] Robin C, Le MP, Melica G, Massias L, Redjoul R, Khoudour N (2017). Plasma concentrations of atovaquone given to immunocompromised patients to prevent Pneumocystis jirovecii. J Antimicrob Chemother.

[167] Sangiolo D, Storer B, Nash R, Corey L, Davis C, Flowers M (2005). Toxicity and efficacy of daily dapsone as Pneumocystis jiroveci prophylaxis after hematopoietic stem cell transplantation: a case-control study. Biol Blood Marrow Transplant.

[168] Bozzette SA, Finkelstein DM, Spector SA, Frame P, Powderly WG, He WL (1995). A randomized trial of 3 antipneumocystis agents in patients with advanced human-immunodeficiency-virus infection. New Engl J Med.

[169] Beumont MG, Graziani A, Ubel PA, MacGregor RR (1996). Safety of dapsone as Pneumocystis carinii pneumonia prophylaxis in human immunodeficiency virus-infected patients with allergy to trimethoprim/sulfamethoxazole. Am J Med.

[170] Vasconcelles MJ, Bernardo MV, King C, Weller EA, Antin JH (2000). Aerosolized pentamidine as pneumocystis prophylaxis after bone marrow transplantation is inferior to other regimens and is associated with decreased survival and an increased risk of other infections. Biol Blood Marrow Transplant.

[171] Khalaf AM, Hashim MA, Alsharabati M, Fallon K, Cure JK, Pappas P (2017). Late-onset cerebral toxoplasmosis after allogeneic hematopoietic stem cell transplantation. The American journal of case reports.

[172] Busemann C, Ribback S, Zimmermann K, Sailer V, Kiefer T, Schmidt CA (2012). Toxoplasmosis after allogeneic stem cell transplantation--a single centre experience. Ann Hematol.

[173] Tomonari A, Takahashi S, Ooi J, Tsukada N, Konuma T, Kato S (2008). No occurrence of Pneumocystis jiroveci (carinii) pneumonia in 120 adults undergoing myeloablative unrelated cord blood transplantation. Transpl Infect Dis.

[174] Konishi M, Yoshimoto E, Takahashi K, Uno K, Kasahara K, Murakawa K (2003). Aerosolized pentamidine prophylaxis against AIDS-related Pneumocystis carinii pneumonia and its short- and long-term effects on pulmonary function in the Japanese. J Infect Chemother.

[175] Obaji J, Lee-Pack LR, Gutierrez C, Chan CK (2003). The pulmonary effects of long-term exposure to aerosol pentamidine: a 5-year surveillance study in HIV-infected patients. Chest..

[176] Wei CC, Pack LL, Chan CK (1998). Effects of long-term aerosol pentamidine for Pneumocystis carinii prophylaxis on pulmonary function. Chest..

[177] Tullis E, Yu DG, Rawji M, Rachlis A, Hyland R, Chan CK (1992). The long-term effects of aerosol pentamidine on pulmonary function. The Toronto Aerosolized Pentamidine Study (TAPS) Group. Clin Invest Med.

[178] Camus F, de Picciotto C, Lepretre A, Landman R, Girard PM (1991). Pulmonary tolerance of prophylactic aerosolized pentamidine in human immunodeficiency virus-infected patients. Chest..

[179] O’Riordan TG, Smaldone GC (1992). Exposure of health care workers to aerosolized pentamidine. Chest..

[180] Balmes JR, Estacio PL, Quinlan P, Kelly T, Corkery K, Blanc P (1995). Respiratory effects of occupational exposure to aerosolized pentamidine. J Occup Environ Med.

[181] Hughes WT, Kuhn S, Chaudhary S, Feldman S, Verzosa M, Aur RJ (1977). Successful chemoprophylaxis for Pneumocystis carinii pneumonitis. N Engl J Med.

[182] Hughes WT (1987). Pneumocystis carinii pneumonitis. N Engl J Med.

[183] Olsen SL, Renlund DG, O'Connell JB, Taylor DO, Lassetter JE, Eastburn TE (1993). Prevention of Pneumocystis carinii pneumonia in cardiac transplant recipients by trimethoprim sulfamethoxazole. Transplantation..

[184] Schneider MM, Nielsen TL, Nelsing S, Hoepelman AI, Eeftinck Schattenkerk JK, van der Graaf Y (1995). Efficacy and toxicity of two doses of trimethoprim-sulfamethoxazole as primary prophylaxis against Pneumocystis carinii pneumonia in patients with human immunodeficiency virus. Dutch AIDS Treatment Group. J Infect Dis.

[185] Hirschel B, Lazzarin A, Chopard P, Opravil M, Furrer HJ, Ruttimann S (1991). A controlled study of inhaled pentamidine for primary prevention of Pneumocystis carinii pneumonia. N Engl J Med.

[186] Link H, Vohringer HF, Wingen F, Bragas B, Schwardt A, Ehninger G (1993). Pentamidine aerosol for prophylaxis of Pneumocystis carinii pneumonia after BMT. Bone Marrow Transplant.

[187] Lim MJ, Stebbings A, Lim SJ, Foor K, Hou JZ, Farah R (2015). IV pentamidine for primary PJP prophylaxis in adults undergoing allogeneic hematopoietic progenitor cell transplant. Bone Marrow Transplant.

[188] Curi DA, Duerst RE, Badke C, Bell J, Chaudhury S, Kletzel M (2016). IV pentamidine for Pneumocystis jiroveci pneumonia prophylaxis in pediatric allogeneic stem cell transplant patients. Bone Marrow Transplant.

[189] Sweiss K, Anderson J, Wirth S, Oh A, Quigley JG, Khan I (2018). A prospective study of intravenous pentamidine for PJP prophylaxis in adult patients undergoing intensive chemotherapy or hematopoietic stem cell transplant. Bone Marrow Transplant.

