# Supplementary material for: Primary prophylaxis of bacterial infections and Pneumocystis jirovecii pneumonia in patients with hematologic malignancies and solid tumors: 2020 updated guidelines of the Infectious Diseases Working Party of the German Society of Hematology and Medical Oncology (AGIHO/DGHO)
Source: Ann Hematol. 2021 Apr 13;100(6):1603–20. doi: 10.1007/s00277-021-04452-9 (PMC8116237; doi:10.1007/s00277-021-04452-9)
Supplement: Supplementary file 1 — (DOCX 23 kb) [file 277_2021_4452_MOESM1_ESM.docx]

# Guideline report: Primary prophylaxis of bacterial infections and Pneumocystis jirovecii pneumonia in patients with hematologic malignancies and solid tumors: 2020 updated guidelines of the Infectious Diseases Working Party of the German Society of Hematology and Medical Oncology (AGIHO/DGHO)

# Reasons for writing this guideline

Bacterial infections and Pneumocystis jirovecii pneumonia (PcP) increase morbidity and mortality in hematologic and oncologic patients with neutropenia. To prevent febrile neutropenia and infections the administration of antibiotic prophylaxis is widely implemented into clinical routine. As global resistance rates are increasing,g rational usage of antibiotics is of utmost importance. This guideline gives recommendations on indications and current strategies for adequate prophylactic treatment to avoid inappropriate administration of antibiotics. Moreover, we intend to discuss the impact of antibacterial prophylaxis on growing resistance rates with regard to antibacterial prophylaxis and human microbiota composition.

We updated the latest version of this guideline to give an overview about current evidence on antibacterial prophylaxis strategies and the choice of drugs to prevent bacterial infection and Pneumocystis pneumonia (PcP) in neutropenic patients. In addition, we revisited patient risk stratification, bacterial epidemiology, and considered the current discussion on potential adverse effects caused by antibiotic exposure. As part of the DGHO, the Infectious Diseases Working Party in Haematology and Medical Oncology (Arbeitsgruppe für Infektionen in der Hämatologie und Onkologie, AGIHO) specializes in guidelines on infection-related issues in Haematological and Oncological patients. In the past, numerous guidelines have been published as a result of this cooperation.

# Intended audience

This guideline appeals to:

- Haematologists / oncologists and other physicians prescribing anti-cancer chemotherapy in hospital or private practice (primary target group)
- Other physicians, nursing, and service staff caring for patients under treatment for malignant diseases
- Patients, policy makers, and insurance companies as a guidance for best practice

# Aim of this guideline

This guideline provides evidence-based recommendations on indications, strategies and therapeutic options for antibiotic prophylaxis in patients at risk for neutropenia. Moreover, the impact of new immune-modulating drugs on development of febrile neutropenia and infections will be emphasized. In the context of globally increasing bacterial resistance rates the justification for antibiotic prophylaxis as well as new aspects regarding the human microbiome will be discussed. This guideline aims to improve patient care through supporting reliable identification of patients at risk and encouraging a rational usage of antibiotics.

# Composition and consensus process of the expert panel

Membership in the AGIHO is free for all DGHO members. The DGHO is the largest German Haematological association. Membership status can be achieved by receiving two recommendations by existing members (peers). There are multiple annual meetings of the AGIHO; members are invited at least four weeks ahead. A full agenda of these meetings is part of the invitation, so all members are informed of guidelines brought up for discussion. The discussion is open for all members and every member present has one vote, with the limitation that only physicians in patient care are allowed to vote. Employees of pharmaceutical companies are excluded from voting.

Consensus for this guideline was achieved by multiple meetings of the AGIHO. First, evidence tables were presented and the expert panel ranked levels of evidence. The team of authors then drafted recommendations based on the evidence grading and the associated discussions. These recommendations were again presented to the panel for further intensive discussion and final decision on the grading and exact wording of the recommendation. If unanimity could not be achieved through discussion, decisions were made by simple majority vote of the panel. Evaluation of strength of recommendation and quality of evidence was performed in consistence with other recently updated German and European guidelines (**Table 1**). In cases with conflicting study results, recommendations were downgraded from A to B. Recommendations based on analogies were only allowed in the absence of sufficient primary data.

**Table 1. Grading system**

| Category, grade | Definition |
| --- | --- |
| Strength of recommendation | |
| A | Strongly supports a recommendation for use |
| B | Moderate evidence to support a recommendation for use |
| C | Marginally supports a recommendation for use |
| D | Supports a recommendation against use |
| Quality of evidence - level | |
| I | Evidence from at least one properly designed randomized, controlled trial |
| II | Evidence from at least one well-designed clinical trial, without randomization; from cohort or case-controlled analytic studies (preferably from > 1 center); from multiple time series; or from dramatic results of uncontrolled experiments |
| III | Evidence from opinions of respected authorities; based on clinical experience; descriptive case studies; or reports of expert committees |
| Quality of evidence – index (for Level II) | |
| r | Meta-analysis or systematic review of randomized controlled trials |
| t | Transferred evidence, that is, results from different patients‘ -cohorts, or similar immune-status situation |
| h | Comparator group is a historical control |
| u | Uncontrolled trial |
| a | Published abstract (presented at an international symposium or meeting) |

# Eligibility criteria and literature search strategy

We included trials evaluating the impact of antibacterial prophylaxis, the choice of antibacterial drugs and the impact of new immune-modulating treatment strategies. Trials evaluating patients with allogeneic stem cell transplantation only were excluded from this review. We preferably evaluate randomized controlled trials (RCTs). If RCTs were not available, data from non-randomised studies were included. Abstracts and preliminary reports were excluded. Trials evaluating treatment strategies in comparable patient populations were included but grading was indexed with an additional mark (s. **Table 1**).

Literature search included publications from January 2012 to August 2020 and was conducted in subcommittees of the expert panel. Selected literature was then discussed in telephone conferences and electronic correspondence by the whole guideline group. Literature was identified by searching the MEDLINE library. If publications were not available in English, French, or German they were excluded from the evaluation process.

All studies included into grading are presented in the supplementary evidence tables and listed in the following order: risk factors, indication for antibacterial prophylaxis, timing and duration of antibacterial prophylaxis, drugs for antibacterial prophylaxis, indication and drugs for P. jirovecii prophylaxis.

# Dissemination strategy

# Copyright for this guideline will be provided to *Annals Of Hematology.* We intend to publish the guideline as Open Access with financial support by the DGHO. Hyperlinks to the publication will be provided on the guideline websites of the DGHO (<http://www.dgho-onkopedia.de>) and the AGIHO (<http://www.dgho-infektionen.de>).

# Updates

Urgent updates will be announced on <http://www.dgho-onkopedia.de> and – if possible – on the homepage of the *Annals Of Hematology*.
